# Comment on “Assessment of Bone Mineral Density in Male Patients with Chronic Obstructive Pulmonary Disease by DXA and Quantitative Computed Tomography”

**DOI:** 10.1155/2017/7370368

**Published:** 2017-05-30

**Authors:** Jose M. Moran, Juan D. Pedrera Zamorano

**Affiliations:** Metabolic Bone Diseases Research Group, Nursing Department, University of Extremadura, Caceres, Spain

We have read with interest the manuscript from Fountoulis et al. [1]. We would like to express a major concern about the statistical analysis presented in the paper.

Data presented in Table 2 was analyzed by means of chi-square test with Monte Carlo simulation when appropriate. The use of the chi-square test for goodness of fit has three assumptions: (i) observations are independent, (ii) categories are mutually exclusive, and (iii) categories are exhaustive [2]. The data presented at minimum fails in the assumption of mutual exclusive categories as a number of patients surely were classified normal, osteopenic, or osteoporotic with both DXA and QCT. Particularly, data is not mutually exclusive as the overall count is 74, which exceeds 37, the number of patients who participated in the study; it means that necessarily some patients were classified in two categories, so the chi-square test for goodness of fit cannot be used.

Additionally, if chi-square test is accepted as a method to analyze the data from Table 2, there is a major concern related to the significance shown in the osteoporotic patients (*P* = 0.04) as the correct *P* value in such case (*n* = 9 patients for DXA and *n* = 16 for QCT) is *P* = 0.230 (nonsignificant), so the data shows that no significant differences are shown between patients diagnosed of osteoporosis by either DXA or QCT. Anyway, we would like to indicate that the application of this statistical procedure in such sample is incorrect.

Based on the data presented by the authors and their analysis, although a great effort was done, it cannot be concluded that the prevalence of low BMD in patients with COPD was higher by using QCT compared to DXA.

## Conflicts of Interest

The authors declared no conflicts of interest.
